# LwPTL: a novel classification for upper airway collapse in sleep endoscopies^☆^

**DOI:** 10.1016/j.bjorl.2019.01.010

**Published:** 2019-03-08

**Authors:** Ahmed Elsobki, Michel Burihan Cahali, Mohamed Kahwagi

**Affiliations:** a University Mansoura, Department of Otorhinolaryngology, Mansoura, Egypt; b Universidade de São Paulo (USP), Hospital das Clínicas, Departamento de Otorrinolaringologia, São Paulo, SP, Brazil

**Keywords:** Pharynx, Epiglottis, Tongue, Endoscopy, Faringe, Epiglote, Língua, Endoscopia

## Abstract

**Introduction:**

There is no consensus on a single classification system for the obstructive findings in drug-induced sleep endoscopy. Previous classification systems have neglected to address the upper retropalatal obstruction, the segmental division of the lateral pharyngeal wall and the primary or secondary nature of laryngeal collapse.

**Objective:**

To propose, illustrate and evaluate a more comprehensive and yet simple classification for drug-induced sleep endoscopy findings.

**Methods:**

Cross sectional study in a tertiary sleep surgery unit. A total of 30 patients with obstructive sleep apnea underwent drug-induced sleep endoscopy according to a new classification system called LwPTL, and its findings were analyzed according to obstructive sleep apnea severity and body mass index. LwPTL incorporates the description of upper retropalatal collapse, distinguishes the lateral pharyngeal wall collapse into three levels and clarify when laryngeal collapses are primary or secondary.

**Results:**

93.3% of the patients presented lateral pharyngeal wall collapse, usually at the level of the velum (73.3%). 80% presented multilevel collapse. Regarding the upper retropalatal region, LwPTL identified 10% of the cases presenting lateral salpingopharyngeal obstruction and 6.6% with high palatal collapse. 3.3% presented epiglottic collapse. Patients presenting lower levels of collapse, either from the lateral wall and/or tongue and/or larynx, represented 30% of the cases and had significantly more severe obstructive sleep apnea, without significant differences in body mass indexes.

**Conclusion:**

LwPTL seems a simple and straightforward staging system for classifying drug-induced sleep endoscopy, distinguishing the important upper retopalatal obstruction and the primary and secondary laryngeal collapses, providing more information for appropriate treatment selection.

## Resumo

**Introdução:**

Não há consenso sobre um sistema único de classificação para os achados obstrutivos da endoscopia do sono induzido por drogas. Os sistemas de classificação anteriores negligenciaram a abordagem da obstrução retropalatal alta, a divisão segmentar da parede lateral da faringe e a natureza primária ou secundária do colapso laríngeo.

**Objetivo:**

Propor, ilustrar e avaliar uma classificação mais abrangente e simples para os achados da endoscopia do sono induzido por drogas.

**Método:**

Estudo transversal em uma unidade terciária de cirurgia do sono. Foram submetidos à endoscopia do sono induzido por drogas 30 pacientes com apneia obstrutiva do sono, de acordo com um novo sistema de classificação denominado LwPTL, e seus achados foram analisados de acordo com a gravidade da apneia obstrutiva do sono e índice de massa corpórea .O LwPTL incorpora a descrição do colapso retropalatal alto, distingue o colapso da parede faríngea lateral em três níveis e esclarece quando os colapsos laríngeos são primários ou secundários.

**Resultados:**

Apresentaram colapso da parede faríngea lateral 93,3% dos pacientes, geralmente no nível do palato mole (73,3%). Em 80% dos pacientes o colapso foi multinível. Em relação à região retropalatal alta, o LwPTL identificou 10% dos casos com obstrução salpingo-faríngea lateral e 6,6% com colapso alto do palato. Em 3,3% ocorreu colapso epiglótico. Pacientes com colapso em regiões baixas, tanto da parede lateral e/ou língua como e/ou laringe, representaram 30% dos casos e apresentaram apneia obstrutiva do sono significativamente mais grave, sem diferenças significantes no índice de massa corpórea.

**Conclusão:**

O LwPTL parece um sistema de estadiamento simples e direto para classificar os achados da endoscopia do sono induzido por drogas, distingue a importante obstrução retropalatal alta e os colapsos laríngeos primários e secundários, fornece mais informações para uma seleção adequada do tratamento.

## Introduction

Obstructive sleep apnea (OSA) is a multifactorial disease. Multiple pathophysiological mechanisms contribute to airway collapse including anatomical narrowing of the upper airway, poor upper airway dilator muscle activity, low arousal threshold and ventilatory control instability.^1^ The pharyngeal airway collapses when inspiratory transpharyngeal pressure exceeds the action of the pharyngeal dilator muscles.^2^ Upper airway collapse may occur in four sites; soft palate, tongue, lateral pharyngeal walls and the larynx.^3^

Considering the wide variety of surgical procedures currently available for treating chronic snoring and OSA, the assessment of the site(s) of upper airway obstruction is important for selecting the appropriate procedure. Multiple evaluation techniques have been developed to examine an individual's pattern of upper airway obstruction.^4^ Drug-induced sleep endoscopy (DISE) has been introduced by Croft and Pringle in 1991.^5^ Today, DISE represents the most widespread diagnostic tool for upper airway endoscopic evaluation of snoring and OSA.^6^ All available DISE classification systems are based on anatomical information only^7^ and, in spite of that, the European Position meeting on DISE has reached no consensus on a scoring and classification system for DISE findings.^6^

Previous classification systems have neglected to address the upper retropalatal obstruction, the segmental division of the lateral pharyngeal wall and the detailed structural pattern of primary or secondary laryngeal collapse. In order to address these issues, we aim to propose, illustrate and evaluate a more comprehensive and yet simple classification for DISE findings, which we called LwPTL.

## Methods

After becoming experienced with both DISE technique and our proposed classification system, we have collected data on the last 30 consecutive OSA patients that underwent DISE and upper airway surgery at the Mansoura University Hospital, a tertiary referral sleep surgery unit in Egypt.

All patients underwent level 1 polysomnography preoperatively (transcutaneous pulse oximetry was used to monitor oxygen saturation and heart rate. The sleep architecture was recorded using electroencephalogram, electro-oculogram and submental electromyogram. We measured thoracic and abdominal effort, movements of limbs, oronasal airflow, and a sensor for snoring). The severity of OSA is expressed in the apnea–hypopnea index (AHI). Obstructive apneas were defined as cessation of airflow for at least 10 s. Hypopneas were defined as periods of reduction of >30% in oronasal airflow for at least 10 s associated with a >4% decrease in oxygen saturation. The AHI was calculated as the sum of total events (apneas and hypopneas) per hour of sleep.

All patients had preoperative DISE for assessment of obstruction levels. We applied our new staging system for DISE findings and correlated the data with the OSA severity and body mass index (BMI).

### Inclusion criteria

Age from 18 to 50 years old;
Available level one sleep study;
OSA patients with AHI > 5 with CPAP failure or refusal.

### Exclusion criteria

Previous sleep surgery;
Pregnancy;
Obvious craniomaxillofacial anomalies.

### DISE technique

For our DISE technique, the patient was in a supine position on the operating table. The patients had basic cardiorespiratory monitoring (pulse oximetry, blood pressure, and electrocardiogram). The target depth of sedation is the transition from consciousness to unconsciousness (loss of response to verbal stimulation), in practical terms, when the patient started to snore and choke. This description for the desired level of sedation is because we do not have the bispectral index monitoring in our department. Atropine was used for all patients once 30 min before anesthesia at a dose of (0.6 mg/kg) as it is anticholinergic drug which decreases saliva during evaluation. Sleep was induced using propofol in a dose of (1.5 mg/kg) as a bolus and then maintained with simply manual controlled infusion. Propofol is an ultra-short acting hypnotic that enables greater control of the depth of sedation during sleep endoscopy. So, slow stepwise induction was used to avoid oversedation. Deeper levels of sedation are associated with progressive decreases in both upper airway dilator muscle tone and neuromuscular reflex activation, which increase airway collapsibility. To avoid overestimation of airway collapse, collapses that occurred while oxygen saturation was less than the minimal saturation in the polysomnography were disregarded.

Once the patient has reached a satisfactory level of sedation, a flexible endoscope lubricated with lidocaine 2% gel was introduced into the nasal cavity. The lateral pharyngeal wall, soft palate, tongue base and larynx were observed. The sites generating snoring and/or obstruction were assessed.

### LwPTL staging system

Our experience with DISE examination suggested that this classification could guide the management protocol. According to data gained from DISE we classified airway collapse into four levels: Lateral pharyngeal Wall (LW), Palate (P), Tongue (T) and Larynx (L) (Table 1). In all levels, we get qualitative information only, i.e., the collapse is either present or absent. Collapse here means total or near total visual obstruction.

**Table 1:** The LwPTL classification for DISE.

Structure	Pattern			Comment
Lateral wall (Lw)	LS	LV	LH	LS (Lw at salpingopharyngeal folds)
				LV (Lw at velum)
				LH (Lw at hypopharynx)
				Combinations (LSV, etc.)
				Lw0: no collapse
Palate	PH	PL		PHL: (high palate collapse) vertical palate
				PL: (low palate collapse) oblique palate
				P0: no collapse
				Never PH (high palate collapse) only
Tongue base	TH	TL		TH: high tongue base collapse
				TL: low tongue base collapse
				THL: both
				T0: no tongue collapse
Larynx	L0	L1		L1: laryngeal collapse
				L0: no collapse
Special tests	Trans oral DISE/positional DISE/Emrich maneuver			

#### Lateral pharyngeal wall collapse: (Lw)

**Figure 1:**
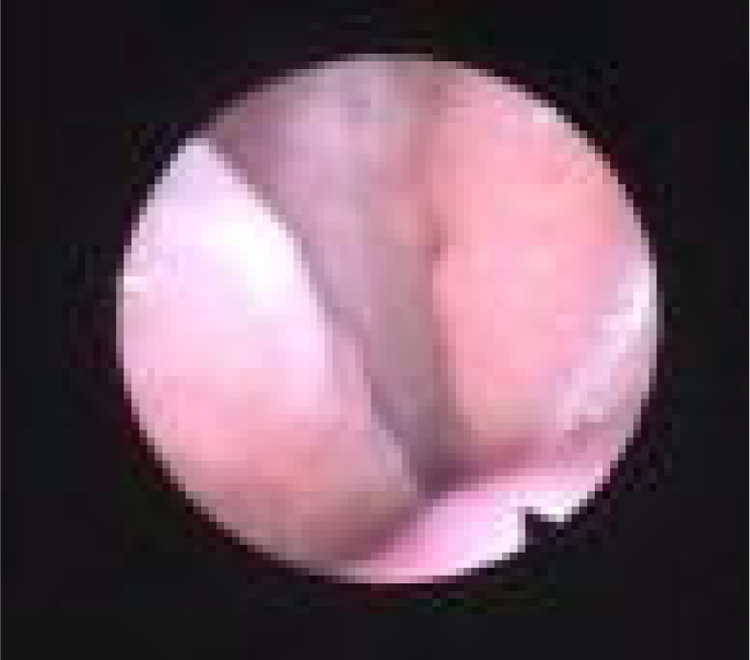
Fiberoptic view of LS collapse, Lateral pharyngeal wall collapse at the level of the salpingeopharyngeal folds.

**Figure 2:**
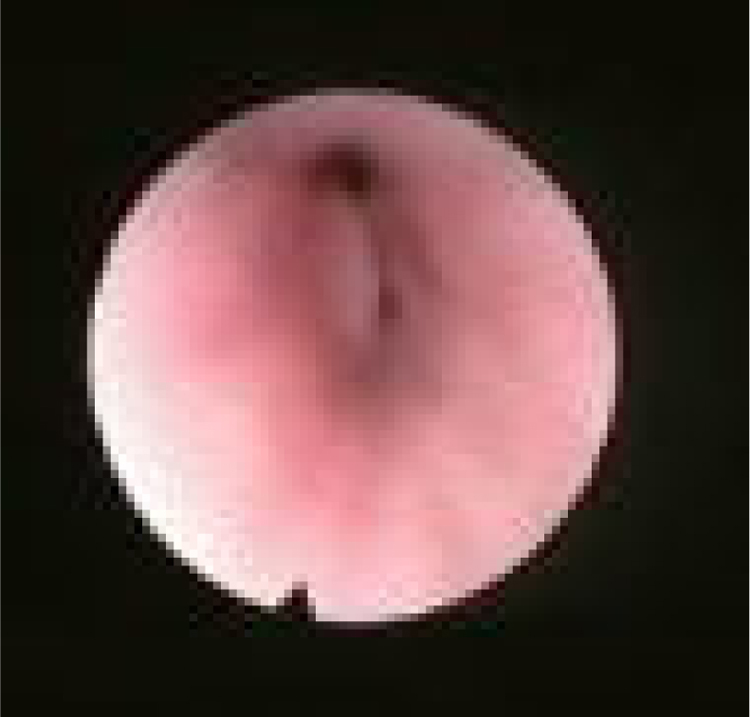
Fiberoptic view of LV collapse, Lateral pharyngeal wall collapse at the level of the velum.

**Figure 3:**
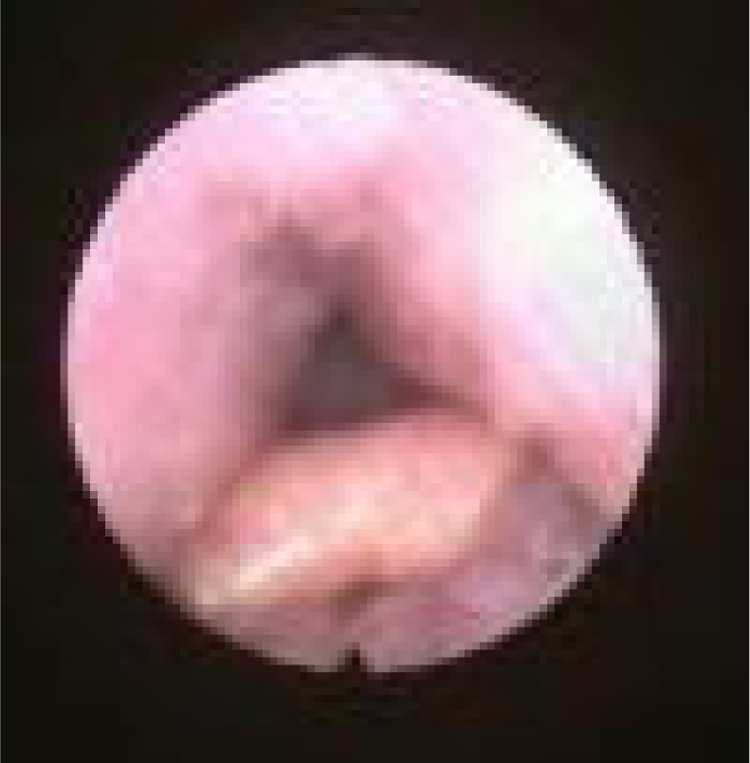
Fiberoptic view of LH collapse, Lateral pharyngeal wall collapse at the level of the hypopharynx.

We subclassified it into collapse at the Level of the Salpingeopharyngeal folds (LS) (Fig. 1), of the Velum (LV) (Fig. 2) and of the Hypopharynx (LH) (Fig. 3). Combination of 2 or 3 of these levels may occur (LSV, LVH and LSVH). LS means that the collapse occurs at the level of the salpingeopharyngeal folds while the velum is patent. Although this pattern is infrequent, it seems important to identify it to avoid unnecessary palatal surgery because this patient may respond to just fold reduction. LV means a lateral wall collapse at the level of the velar segment of the soft palate not caused by hypertrophic folds; any tonsillar level collapse is also classified as LV. LH means collapse of the lateral pharyngeal wall distal to the tonsils, sometimes causing secondary epiglottic collapse by pushing the epiglottis from side to side.

#### Palatal level of collapse: (P)

**Figure 4:**
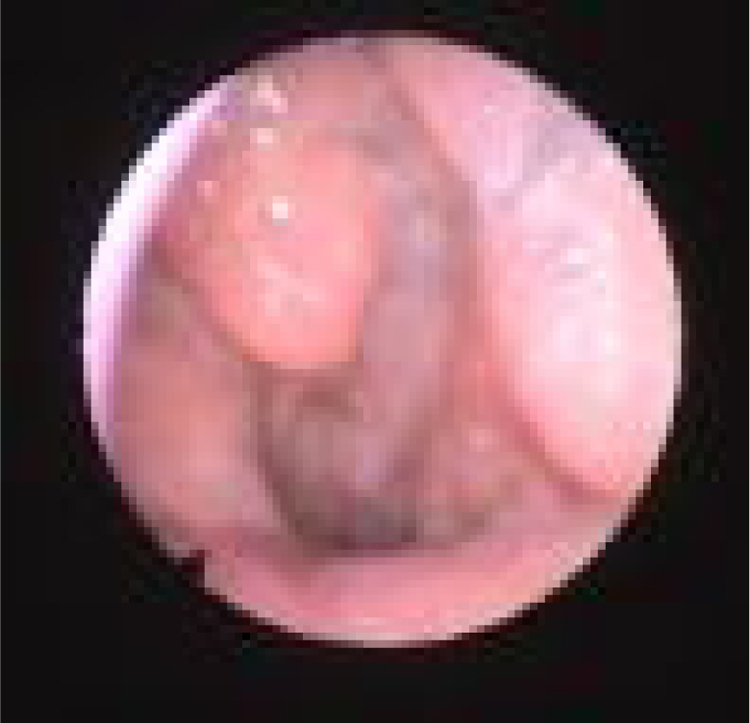
Fiberoptic view of PHL collapse, High palatal collapse.

**Figure 5:**
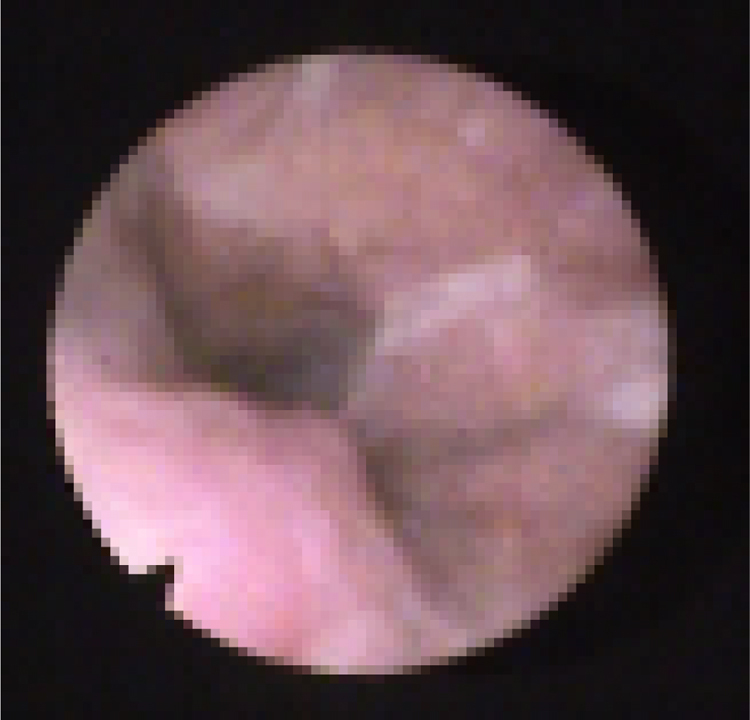
Fiberoptic view of PL collapse, Low palatal collapse.

Palatal collapse refers to anteroposterior collapse only. We classified it according to Woodson classification^8^ into High Palatal Collapse (PHL) (Fig. 4) which means collapse of the muscular and aponeurotic segments of the soft palate (the collapse occurs on both high and low levels so PH alone does not exist) and low Palatal Collapse (PL) (Fig. 5) which means collapse of the distal segment of the soft palate. Although somehow popular description of collapse, we think that circular collapse of the palate is a misleading term, because it brings attention to the palate where we believe the major pathophysiological components of that collapse is the lateral wall. Therefore we preferred not to use the term circular palatal collapse in our classification and instead, individualized the structures involved in that pattern of collapse (LvPl).

#### Tongue base collapse: (T)

**Figure 6:**
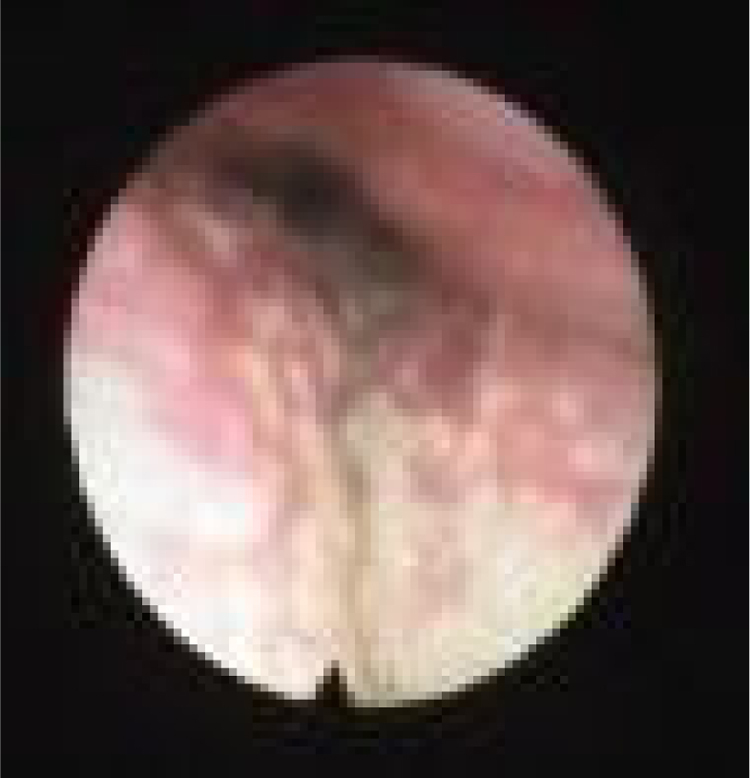
Fiberoptic view of TH collapse, High tongue collapse.

**Figure 7:**
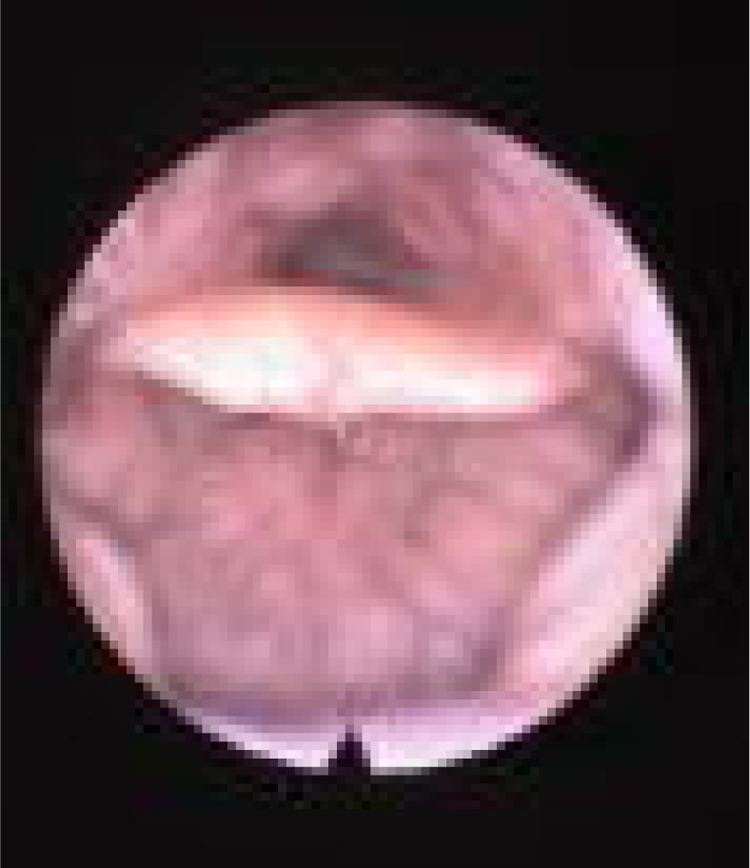
Fiberoptic view of TL collapse, low tongue collapse causing secondary epiglottic collapse.

We classified tongue base collapse into High Tongue base collapse (TH) (Fig. 6) which means collapse of the oropharyngeal tongue (above the level of epiglottis) and low tongue base collapse (at or below the level of epiglottis) (TL) (Fig. 7) which means collapse of the hypopharyngeal tongue causing secondary epiglottic collapse.

#### Laryngeal collapse: (L)

**Figure 8:**
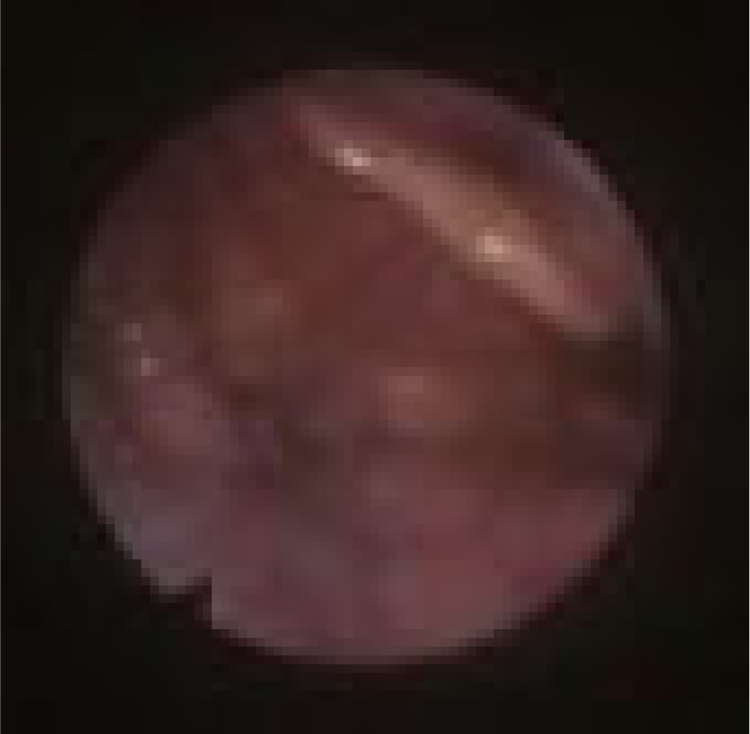
Fiberoptic view of Epiglottic collapse (Floppy epiglottis).

**Figure 9:**
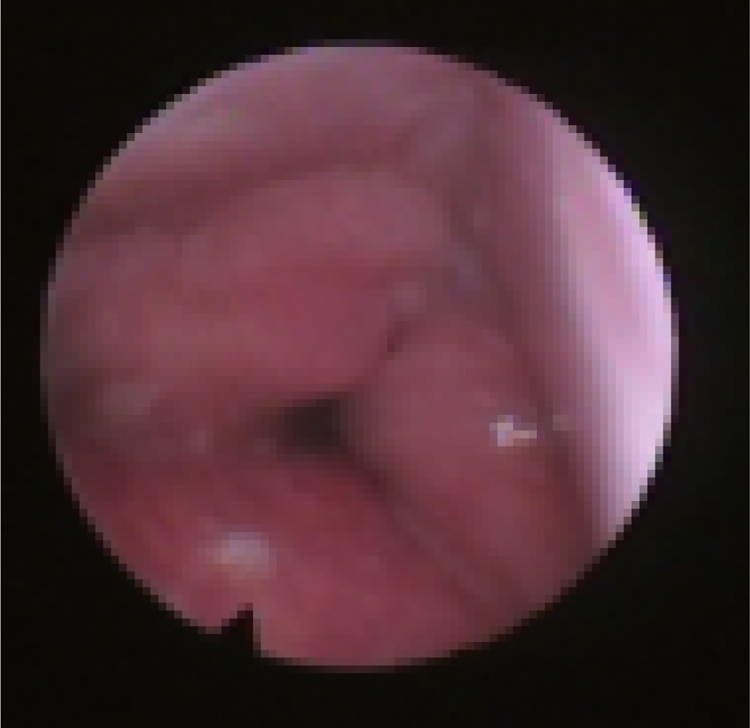
Fiberoptic view of Arytenoid collapse.

Any site of laryngeal collapse not caused by low tongue base collapse or lateral wall hypopharyngeal collapse is put under category Larynx (L) including epiglottic (Fig. 8), aryepiglottic or arytenoid collapse (Fig. 9). Primary laryngeal collapse means that the larynx collapses on its own like the situation in floppy epiglottis while secondary collapse means that neighboring structures like base tongue and lateral wall hypopharynx push on the laryngeal inlet while collapsing.

#### Special tests

**Figure 10:**
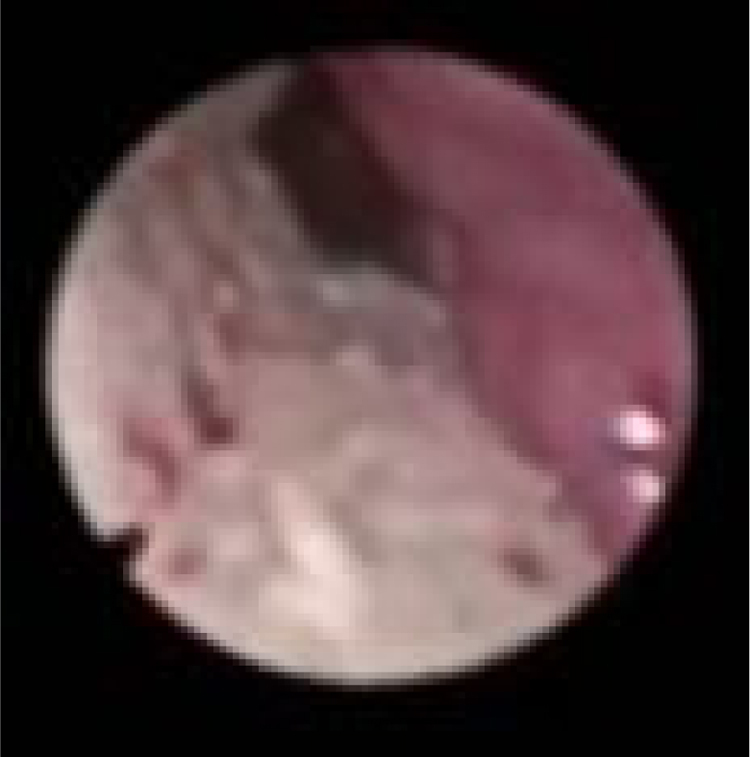
Fiberoptic view of transoral DISE showing the oral tongue not pushing the palate (negative tongue palate interaction).

**Figure 11:**
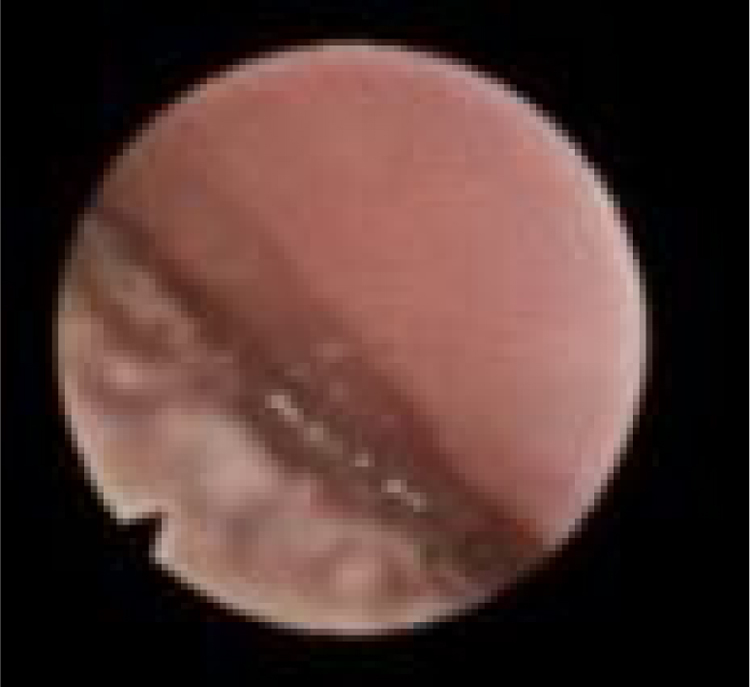
Fiberoptic view of transoral DISE showing the oral tongue pushing the palate (positive tongue palate interaction).

Special tests were done when needed; we performed positional DISE by rotating the head to one side. In addition, transoral DISE was done to assess the tongue palate interaction (sometimes the palate is being pushed by the oral tongue, which might need correction) (Figure 10, Figure 11). During transoral DISE the endoscope is smoothly pushed between the upper and lower incisors with no need to open the mouth to avoid the effect of mouth breathing on upper airway structures. In addition, jaw thrust is performed during DISE to detect the possible benefit of a mandibular advancement device.

**Figure 12:**
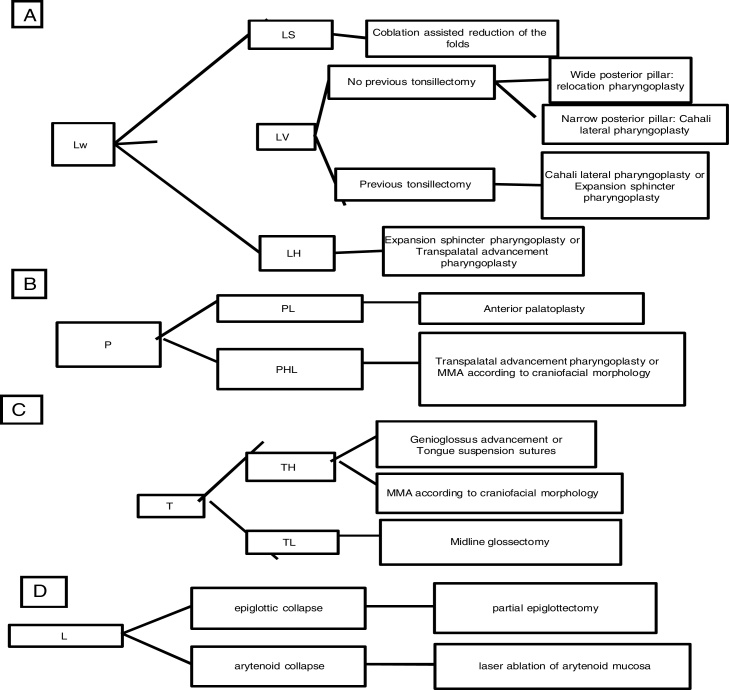
Brief schematic presentation for surgical approach used according to classification system: (A) for Lw, lateral pharyngeal wall collapse and subdivisions; (B) for P, Palatal collapse and subdivisions; (C) for T, tongue collapse and subdivisions; (D) for L, laryngeal collapse.

According to the staging system summarized in Table 1, the first author suggests this classification to guide the surgical procedure selection according to the scheme in Fig. 12.

The rationale for using transpalatal advancement pharyngoplasty for treating the lateral pharyngeal wall collapse at the hypopharyx is to benefit from the anterior traction of the soft palate to increase the tension at the lateral pharyngeal walls more distally.

### Statistical analysis

Data was analyzed using (Statistical Package for Social Sciences) SPSS version 15. Quantitative normally distributed data was presented as mean ± SD. Comparison of continuous data like AHI and BMI as regard to each level of collapse being present or absent was done using Student *t*-test and Mann–Whitney test; *p*-value < 0.05 was considered to be statistically significant.

## Results

After analyzing the data gained from DISE, we could find the following in the 30 patients with median AHI (31.72 ± 20.39 events/h) and mean BMI of (32.97 ± 2.77 kg/m^2^).

Only 11 patients (36.6%) had one level of collapse and the other 19 patients (63.3%) had multilevel collapse. The presence of the multilevel pattern, compared to those with single level collapse, implied in a significant increase in AHI (*p* = 0.003), without significant differences in BMI (*p* = 0.50) (Table 2).

**Table 2:** Statistical analysis of the difference in Apnea–Hypopnea Index (AHI) and Body Mass Index (BMI) between patients with multilevel collapse and those with single level of collapse.

Levels	AHI (events/h)	BMI (kg/m^2^)
Multilevel (*n* = 19)	37.9 ± 21.4	33.2 ± 3.5
Single level (*n* = 11)	16.6 ± 5.1	34.3 ± 5.4
*p*-Value	0.003^a^	0.50

Lateral Wall collapse (Lw) was present in 28 patients (93.3%) and this emphasizes our look for the importance of the lateral wall collapse in OSA patients. Segmental classification of lateral wall collapse showed that of those 28 patients we could find 3 patients with LS, 22 patients with LV and 3 patients with LH. The presence of LH pattern, compared to those without LH collapse, implied in a significant increase in AHI (*p* < 0.0001) and in BMI (*p* < 0.002) (Table 3).

**Table 3:** Statistical analysis of the difference in Apnea–Hypopnea Index (AHI) and Body Mass Index (BMI) between patients with LH (lateral wall collapse at the Hypopharynx) and those without LH.

LH collapse	AHI (events/h)	BMI (kg/m^2^)
Negative (*n* = 27)	26.74 ± 14.88	32.48 ± 2.46
Positive (*n* = 3)	72.00 ± 19.70	37.33 ± 1.15
*p*-Value	<0.0001^a^	0.002^a^

According to Woodson classification,^8^ Low Palatal Collapse (PL) was found in 18 patients (60%) while High Palatal Collapse (PHL) was found in only 2 patients (6.6%). Circular collapse at the level of the velum meaning LvPl was encountered in 10 patients (33.3%). The presence of PHL pattern, compared to those without PHL collapse, implied in a significant increase in AHI (*p* = 0.011) (Table 4). The presence of circular collapse at the palate, compared to those without circular collapse, did not imply in any significant difference in AHI or in BMI (Table 5).

**Table 4:** Statistical analysis of the difference in Apnea–Hypopnea Index (AHI) and Body Mass Index (BMI) between patients with PHL (High Palatal Collapse) and those without PHL.

PHL	AHI (events/h)	BMI (kg/m^2^)
Negative (*n* = 28)	28.82 ± 18.81	32.75 ± 2.74
Positive (*n* = 2)	65.50 ± 0.71	36.00 ± 0.00
*p*-Value	0.011^a^	0.111

**Table 5:** Statistical analysis of the difference in Apnea–Hypopnea Index (AHI) and Body Mass Index (BMI) between patients with circular collapse and those without circular collapse.

Circular collapse (PL + LV)	AHI (events/h)	BMI (kg/m^2^)
Negative (*n* = 20)	31.25 ± 22.2	34.5 ± 4.22
Positive (*n* = 10)	32.7 ± 15.05	32.7 ± 3.06
*p*-Value	0.63	0.24

Low Tongue base collapse (TL) was found in 4 patients (13.3%) and High Tongue base collapse (TH) was found in 2 patients(6.6%). Laryngeal collapse was found in only one patient (3.3%), who had primary epiglottic collapse. Patients presenting the lower levels of collapse, either from the lateral wall and/or tongue and/or larynx, represented 30% of the cases and had significantly more severe OSA (*p* = 0.0008), without significant differences in BMI (Table 6).

**Table 6:** Statistical analysis of the difference in Apnea–Hypopnea Index (AHI) and Body Mass Index (BMI) between patients with hypopharyngeal collapse (LH, Lateral Hypopharyngeal wall; TH, High Tongue base; TL, Low Tongue base; L, Larynx), and those without hypopharyngeal collapse.

Low levels (LH, TH, TL, L)	AHI (events/h)	BMI (kg/m^2^)
Negative (*n* = 21)	23.33 ± 12.09	33.09 ± 4.36
Positive (*n* = 9)	51.33 ± 21.4	35.77 ± 1.75
*p*-Value	0.0008^a^	0.087

**Figure 13:**
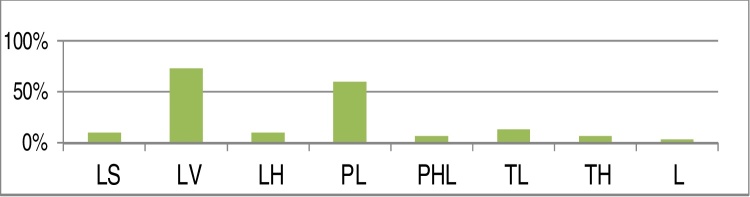
Percent distribution of each level of collapse using the LwPTL classification for DISE (LS, Lateral wall collapse at the level of salpingeopharyngeal fold; LV, Lateral wall collapse at the level of the velum; LH, Lateral wall hypopharyngeal collapse; PL, Low palatal collapse; PHL, High and low palatal collapse; TL, Low tongue base collapse; TH, High tongue base collapse; L, Primary laryngeal collapse).

Fig. 13 summarizes the frequency of the patterns of collapse in our series, using the LwPTL classification for DISE.

## Discussion

There is currently no universally accepted classification system for the data obtained from DISE. Some classification systems prefer to use anatomical levels while others prefer anatomical structures. About 14 classification systems and 3 modifications for the structures that collapse in DISE have been identified till now.^6^, ^7^ Croft and Pringle proposed the first concept of a DISE classification system which consists of three categories.^5^ The proposed classification system was easy to understand. However, it was incomplete for detailed analysis and reporting DISE findings. Since that classification, many appeared with many modifications.^9^, ^10^, ^11^, ^12^, ^13^, ^14^, ^15^, ^16^

The VOTE classification was the first to analyze the data obtained from DISE encompassing the degree and configuration of the obstruction related to the 4 structures (velum, oropharynx, tongue and epiglottis).^12^ However, Kerizian et al. mentioned that the limitation of The VOTE classification was an oversimplification that overlooks interactions between the upper airway structures.^4^

Proper lateral wall splinting is still one of the limitations in sleep surgery. Improving outcomes may arise from a more accurate evaluation. In our view, it is an over simplification to describe the whole lateral pharyngeal wall as a single level of collapse. Lateral wall collapse should be thouroughly evaluated, segmentally classified and accordingly managed, because it does not collapse as one single unit.^17^

Recently we used transoral DISE to look at tongue palate interaction and to see if the oral tongue is pushing the soft palate or not. We could find that there is tongue palate contact in almost all patients. However, this simple contact did not seem to affect the collapsibility of the soft palate. In only one case out of the thirty patients we found a positive tongue palate interaction, meaning that we had the impression that this palate was being pushed by the tongue and, actually, this did not change our procedure selection because tongue palate interaction is not yet taken into account in our treatment protocols. Future follow-ups on cases presenting that feature are important for establishing whether tongue base management is required or not in those cases.

The severity of upper airway collapse in patients with sleep apnea may decrease significantly when the head is rotated to the lateral side. However, there is no significant difference between the rotation of the head to the right or to the left side.^18^ Adding this positional maneuver into DISE may provide important insights regarding where the primary site of obstruction is located.

In our classification, we intentionally did not quantify the degree of obstruction because there is no data showing any physiological meaning for partial visual collapses and usually a partial obstruction does not produce a recognizable decrease in flow,^19^ also, this simplification would potentially improve inter examiner agreement.

Woodson 2015 described 3 patterns for palatal morphology: the oblique palate in which the narrowing is at the velum, the intermediate palate in which the narrowing is at the velum and genu and the vertical palate at which the narrowing is at the velum, genu and the hard palate. These patterns were the rationale for selecting patients for the transpalatal advancement procedure.^8^ Our classification system incorporates that rationale, where PHL implies vertical palate morphology and PL implies an oblique morphology.

The classification of tongue base collapse into high or low would guide the procedure selection, respectively, toward bony framework surgery or tongue base reduction. To avoid overestimation of the laryngeal level of collapse, secondarily pushed epiglottis by low tongue base or lateral hypopharyngeal wall are described with the pushing level.

The European position paper stated that: “hypopharynx has its superior limit at the level of the hyoid bone, where it is contiguous with the oropharynx and the major subsites of the hypopharynx are the pyriform sinuses, the post-cricoid region, and the pharyngeal wall. Therefore, this region is not involved in the collapse”.^20^ We respect the statement, but we also think that everyone should recognize how unlikely it is to identify the hyoid bone during DISE and, therefore, we still look at the hypopharyngeal lateral wall in sleep endoscopic terms, naming it to the most distal part of the lateral pharyngeal wall. It is important to individualize the collapses at that particular level in order to tailor an appropriate surgical treatment for that region. In our sample, collapses generated from structures located at the hypopharynx were associated with significantly higher AHI- but not be BMI compared to those without hypopharyngeal obstruction.

### Limitation of the study

The small number of patients in our study is one limitation for a more robust statistical analysis. Another limitation is that we are not presenting the impact of this staging system on the outcomes of the surgical procedure selected, which is still under investigation and makes room for future studies.

## Conclusion

LwPTL seems a simple and straightforward staging system for classifying DISE, being the first to segmentally evaluate the lateral pharyngeal wall collapse and to distinguish the important upper retropalatal obstruction and to standardize primary and secondary laryngeal collapse. Application of this simplified detailed staging system may help with accurate surgical planning.

## Conflicts of interest

The authors declare no conflicts of interest.
